# Thermal Frequency Reconfigurable Electromagnetic Absorber Using Phase Change Material

**DOI:** 10.3390/s18103506

**Published:** 2018-10-17

**Authors:** Heijun Jeong, Jeong-Heum Park, You-Hwan Moon, Chang-Wook Baek, Sungjoon Lim

**Affiliations:** School of Electrical and Electronics Engineering, Chung-Ang University, 84 Heukseok-ro, Dongjak-gu, Seoul 06974, Korea; jhijun000015@gmail.com (H.J.); meteor9147@gmail.com (J.-H.P.); dptmxps@naver.com (Y.-H.M.)

**Keywords:** electromagnetic absorber, frequency reconfigurable absorber, thermally reconfigurable, phase changing material

## Abstract

In this study, we propose a thermal frequency reconfigurable electromagnetic absorber using germanium telluride (GeTe) phase change material. Thermally-induced phase transition of GeTe from an amorphous high-resistive state to a crystalline low-resistive state by heating is used to change the resonant frequency of the absorber. For full-wave simulation, the electromagnetic properties of GeTe at 25 °C and 250 °C are characterized at 10 GHz under normal incidence for electromagnetic waves. The proposed absorber is designed based on the characterized electromagnetic parameters of GeTe. A circular unit cell is designed and GeTe is placed at a gap in the circle to maximize the switching range. The performance of the proposed electromagnetic absorber is numerically and experimentally demonstrated. Measurement results indicate that the absorption frequency changes from 10.23 GHz to 9.6 GHz when the GeTe film is altered from an amorphous state at room temperature to a crystalline state by heating the sample to 250 °C. The absorptivity in these states is determined to be 91% and 92%, respectively.

## 1. Introduction

Metamaterial is an artificial structure with extraordinary electromagnetic characteristics not found in nature. Since the permittivity and permeability of metamaterials can be artificially tailored, they have been used in many applications such as electromagnetic interface (EMI)/electromagnetic compatibility (EMC) solutions [1], terahertz devices [2,3,4], stealth technologies [5,6,7], acoustic [8,9,10], and human body applications [11]. Especially, electromagnetic absorption is a promising application for metamaterials. In general, high lossy materials have been used to realize electromagnetic absorbers. For instance, a wedge tapered absorber [12,13,14] provides excellent absorptivity using ferrite or composite materials. Despite their excellent absorptivity, wedge tapered absorbers are bulky and the material cost is very high. An alternative candidate is the Jaumann absorber [15,16], which is based on a resonant structure and resistive sheet. Although it can be made thinner than wedge tapered absorbers, a quarter-wavelength (λ/4) thickness for dielectric materials is required. Thinner electromagnetic absorbers were recently created using metamaterials [17,18,19]. These metamaterial absorbers can achieve high absorptivity despite their low substrate thickness. Low-cost and easy fabrication are additional advantages.

Despite these advantages, metamaterial absorbers suffer from narrow absorption bandwidth due to their resonance characteristics. A potential solution to this drawback is broadband metamaterial absorbers or frequency reconfigurable metamaterial absorbers. The former has been developed using resistive ink [20,21], multi resonance [22,23,24,25], or lumped elements [26,27,28] and the latter has been developed using electronic devices [29,30,31,32], fluidically tunable devices [33,34,35,36,37], or mechanically tunable devices [38,39,40]. Electronically reconfigurable metamaterial absorbers using P(Positive)-I(Intrinsic)-N(Negative) diodes [29,30,31] or varactor diodes [32] provide advantages of instantaneous response and compatibility with electronic circuits. However, the overall cost is high because the metamaterial is built as a periodic array and the number of electronic devices is extremely large. Bias lines for electronic devices limit the absorber design and performance. Fluidically reconfigurable metamaterial absorbers using liquid crystal [33,34], ethanol [35], or liquid metal [36,37] provide advantages of no DC power consumption and no complicated bias network. However, slow tuning speed can limit their practical applications. Mechanically reconfigurable metamaterial absorbers such as stretchable absorbers have been developed [38,39,40]. They provide advantages of not only cost-effectiveness but also simple design. However, slow tuning speed and stability are limitations to practical applications.

In this study, we developed a thermal frequency switchable electromagnetic absorber using phase change germanium telluride (GeTe) material. Phase change material (PCM) undergoes a drastic change in resistivity according to its state. For instance, chalcogenide PCMs can thermally transition between crystalline (low resistive, conductive) and amorphous (high resistive, insulating) states. Since chalcogenide PCM can be switched quickly between the two states using pulsed heating methods and does not need to be powered to maintain either state, it has been used as a switching device for non-volatile memory or logic applications [41,42]. Complex metal oxide PCMs such as vanadium dioxide (VO_2_), which has a volatile phase change transition, have been applied to demonstrate tunable metamaterial absorbers at RF/microwave or terahertz (THz) frequencies [22,43,44]. Compared to VO_2_, GeTe is known as a PCM material with a very high ON/OFF resistance contrast ratio and non-volatile phase change transition, thus is a potential switching material for RF applications [45,46,47]. In the present paper, we demonstrate a frequency switchable electromagnetic absorber whose resonant frequency can be altered according to the resistivity of GeTe in the ON/OFF states. The proposed electromagnetic absorber can be potentially used for not only frequency switchable absorption but also wireless thermal shock detecting sensor or protection device applications. The proposed absorber is demonstrated through both full-wave simulation and experimental verification. The proposed material will be explained in detail in the following sections.

## 2. Numerical Simulation

The absorptivity *A*(*ω*) can be calculated with the following Equation (1).

(1) A(ω)=1−Γ(ω)−T(ω) 

where Γ(*ω*) and *T*(*ω*) represent the reflection coefficient and transmission coefficient, respectively. According to Equation (1), Γ(*ω*) and *T*(*ω*) should be minimized to achieve high absorptivity. In addition, Γ(*ω*) under normal incidence is calculated with the following equations.

(2) $$\Gamma (\omega )=\frac{Z_{0}-Z_{M}}{Z_{0}+Z_{M}}\;$$

where Z_0_ is the impedance of the free space and Z*_M_* is the impedance of the absorber.

(3) $$Z_{M}=\sqrt{\frac{\mu_{0}\mu_{r}}{\varepsilon_{0}\varepsilon_{r}}}=\sqrt{\frac{\mu_{r}}{\varepsilon_{r}}}\;$$

where $\varepsilon_{0}$ and $\mu_{0}$ are the permittivity and permeability of the free space and, $\varepsilon_{r}$ and $\mu_{r}$ are the permittivity and permeability of the absorber, respectively. Therefore, to achieve the zero values of reflection coefficient (Γ(*ω*)), the absorber impedance (Z*_M_*) should be equal with the free space impedance of 377 Ω (Z*_M_*) to design the absorber.

**Figure 1:**
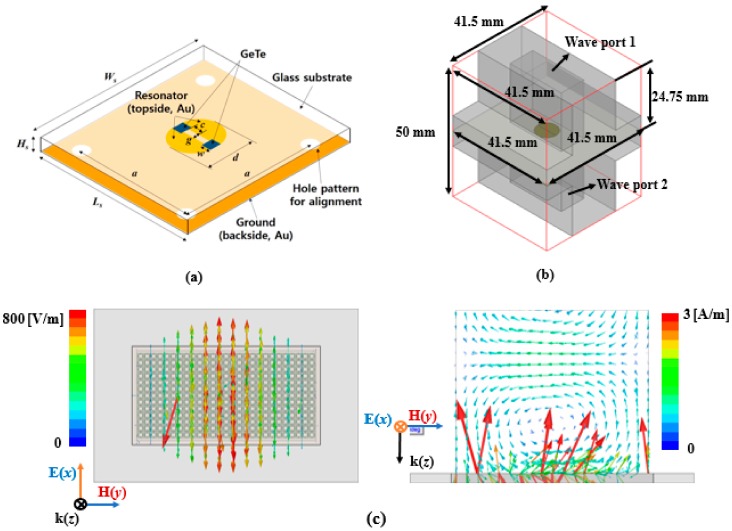
(**a**) Three-dimensional unit cell geometry of the proposed frequency reconfigurable electromagnetic absorber, (**b**) waveguide setup for the numerical simulation and (**c**) simulated E, H- field vectors on XY and YZ planes in the rectangular waveguide at 10.4 GHz.

Figure 1 shows a three-dimensional illustration of the unit cell of the proposed absorber and waveguide setup for the numerical simulations. We use the ANSYS high frequency structure simulator (HFSS) for electromagnetic analysis of the structure. To simulate the overall structure, we designed a pair of waveguides including the proposed absorber as shown Figure 1b. The waveguide dimensions are determined according to the WR-90 waveguide datasheet (WR-90 waveguide size: 41.5 mm × 41.5 mm × 24.75 mm). In order to excite electromagnetic (EM) wave, a pair of wave ports are used in the waveguide as the excitation port as illustrated in Figure 1b. The x-polarized electric field is set in the wave port. We set the radiation box as the air material with 41.5 mm × 41.5 mm × 50 mm size. At the 10.4 GHz, the rectangular waveguide supports a transverse-electric (TE) mode propagating to *z* direction. Therefore, magnetic field is polarized along *y* and *z* directions while electric field is polarized along *x* direction as shown Figure 1c. As shown in Figure 1a, a borosilicate glass wafer is used as a substrate and its dielectric constant (*ɛ_r_*) is determined to be 4.35 from measurements. The loss tangent at 9.6 GHz and 10.5 GHz is 0.05 and 0.03, respectively. A circular conductive gold pattern with a gap in the middle is formed at the top of the substrate while the bottom plane is completely covered by a gold conductor. The GeTe is loaded on the gap of the top plane to alter the resonant frequency.

As-deposited GeTe thin film at room temperature is initially in the amorphous state, and thus has a very high resistivity. Accordingly, the GeTe is in the OFF state, and functions as a capacitor while the gap serves as an open state. On the other hand, when the GeTe is heated above its recrystallization temperature (*T_c_*), phase transition from the amorphous to the crystalline state occurs and the GeTe film has low resistivity. Therefore, the GeTe thin film becomes conductive and functions as an inductor, while the gap in the proposed unit cell serves as a short state. In order to maximize the frequency switching range, two GeTe films are placed at the edges of the gap. Four-hole patterns are created at the edges of the glass to align the absorber sample to the waveguide for measurement. The dimensions of the proposed unit cell are *a* = 31 mm, *c* = 1.6 mm, *d* = 8 mm, *w* = 1.2 mm, *g* = 0.1 mm, *L_s_* = *W_s_* = 41.3 mm, and *H_s_* = 0.5 mm.

**Figure 2:**
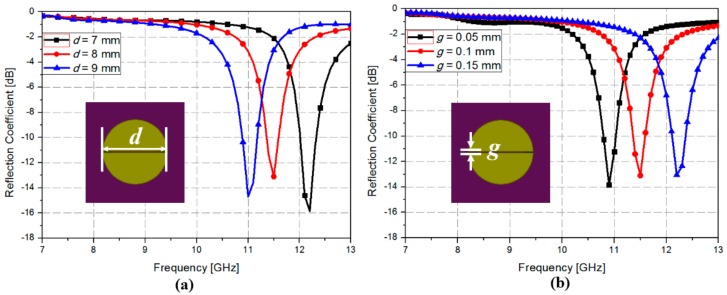
Simulated reflection coefficients without GeTe film when (**a**) *d* is varied from 7 to 9 mm with *g* = 0.1 mm (**b**) *g* is varied from 0.05 to 0.15 mm with *d* = 8 mm.

First, to determine the resonator dimensions, we performed a parametric study with varying dimensions for the resonator using EM simulation. Figure 2 shows the simulated reflection coefficients of the proposed absorber without the GeTe film. As shown in Figure 2a, when the resonator radius (*d*) was increased from 7 to 9 mm with *g* fixed to 0.1 mm, the resonant frequency decreased from 12.2 to 11.0 GHz, respectively. In this study, we chose the resonator radius (*d*) of 8 mm because the WR90 waveguide operates from 8 to 12 GHz. The simulated reflection coefficients were plotted when the resonator gap distance (*g*) was varied from 0.05 to 0.15 mm. When *d* was increased from 0.05 to 0.15 mm, the resonant frequency was increased from 12.2 to 10.9 GHz, respectively, because of lower capacitance. After considering the fabrication limit, a 0.1 mm gap distance was chosen for this study.

**Figure 3:**
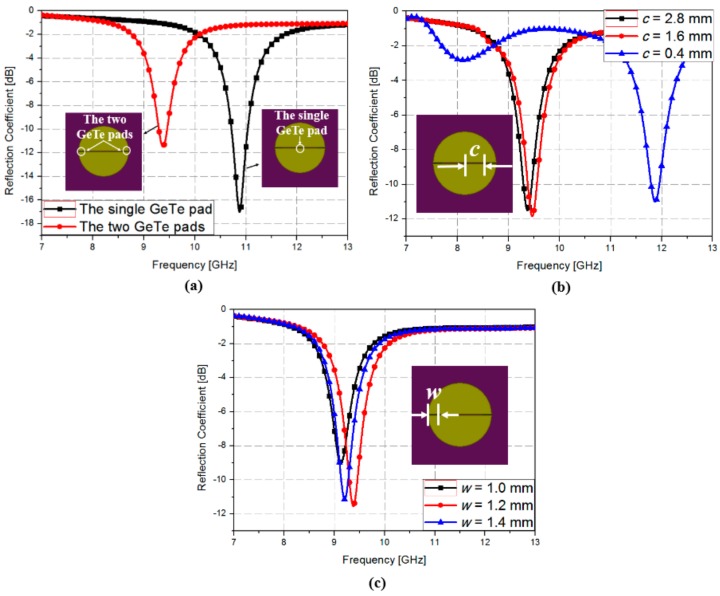
Simulated reflection coefficients of the absorber when GeTe is in the crystalline (ON) state (**a**) with a single GeTe pad and two GeTe pads (**b**) when *c* is varied from 0.4 to 2.8 mm with *d* = 8 mm (**c**) when *w* is varied from 1.0 mm to 1.4 mm with *d* = 8 mm and *g* = 0.1 mm.

Figure 3 shows the parametric simulation results for the GeTe film. In order to achieve the widest switching range, two GeTe films were employed as illustrated in Figure 1. Figure 3a shows the simulated reflection coefficients of the absorber with a single GeTe pad and two GeTe pads when the GeTe is in the crystalline (ON) state. When a single GeTe film was used, the resonant frequency was 10.9 GHz. When two GeTe films were used, the resonant frequency was 9.4 GHz. Therefore, a wider frequency change could be achieved with two GeTe pads. To determine the position of the GeTe film, we simulated the reflection coefficients when the GeTe position (*c*) was varied from 0.4 to 2.8 mm. As shown in Figure 3b, when *c* was 0.4 mm, the resonant frequency did not change. On the other hand, when *c* was 1.6 and 2.8 mm, the resonant frequency was almost similar at 9.45 and 9.5 GHz, respectively. Therefore, we chose 1.6 mm as the GeTe position (*c*). Figure 3c shows the simulated reflection coefficients when the GeTe width (*w*) was varied from 1.0 to 1.4 mm. When the GeTe width (*w*) was 1.2 mm, the reflection coefficient was −11.5 dB. Therefore, to achieve a reflection coefficient less than −10 dB, we chose 1.2 mm as the GeTe width (*w*).

**Figure 4:**
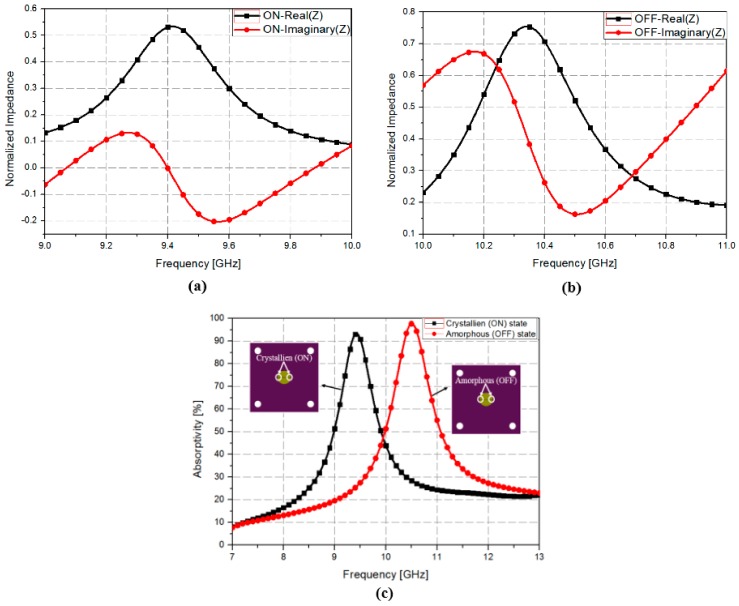
Normalized real and imaginary parts of the impedance of the proposed absorber in the (**a**) crystalline (ON) and (**b**) Amorphous (OFF) states. (**c**) Simulated absorptivity versus frequency for GeTe in different states.

For EM simulation, we needed to model the conductivity of the GeTe film in the amorphous (OFF) and crystalline (ON) states. Firstly, the conductivities of the GeTe were evaluated at DC range by measuring the sheet resistance of the separately deposited GeTe thin film before and after heat treatment at 250 °C. Sheet resistances of the as-deposited and crystallized GeTe thin films were measured to be 120 MΩ/□ and 60 Ω/□, respectively. They corresponded to the conductivities of 8.33 × 10^−2^ S/m and 1.67 × 10^5^ S/m, respectively. Based on these parameters, we initially designed the resonator in the waveguide. In order to characterize conductivities of GeTe at RF range, we measured S-parameters of the fabricated resonator with the GeTe film in the waveguide setup. From the measured S-parameters, the conductivity of the GeTe films in OFF and ON states were modeled as 0.63 × 10^−2^ S/m (high resistivity) and 3.2 × 10^5^ S/m (low resistivity) at 10 GHz, respectively. Figure 4 shows the simulated absorptivity versus frequency for GeTe in different states using these conductivities in ANSYS HFSS.

The absorptivity can be calculated using Equations (1) and (2). Therefore, to achieve more than 90% absorptivity, the absorber impedance should be matched with free space impedance from 0.5 × Z_0_ to Z_0_. Figure 4a,b shows the normalized real and imaginary parts of the impedance of the proposed absorber when the GeTe film is in the crystalline (ON) and amorphous (OFF) states. When the GeTe film is in the crystalline (ON) state, the normalized real and imaginary impedance at 9.4 GHz is 0.55 and 0, respectively. On the other hand, when the GeTe film is in the amorphous (OFF) state, the normalized real and imaginary impedance at 10.5 GHz is 0.52 and 0.15, respectively. As a result, the absorption frequency can be calculated from Figure 4a,b, and the calculated simulation results are shown in Figure 4c. As shown in Figure 4c, the absorption frequency changes from 10.51 GHz with 92% absorptivity to 9.4 GHz with 99% absorptivity when the GeTe transitions from the amorphous state to the crystalline state.

**Figure 5:**
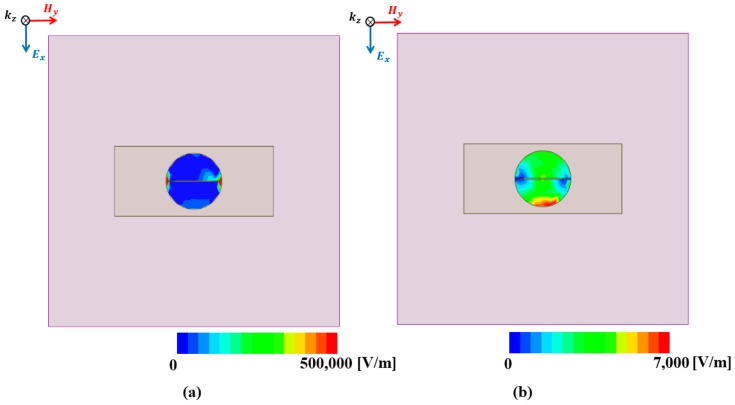
Simulated electric field distribution of the unit cell when the GeTe is in (**a**) the amorphous (OFF) state and (**b**) the crystalline (ON) state.

Figure 5 shows the simulated magnitude of the electric field distribution of the unit cell according to the state of the GeTe material. As shown in Figure 5a, the electric field is distributed in both ends of the gap when the GeTe films are in the amorphous (OFF) state. When the GeTe is heated up and transitions to the crystalline (ON) state, the gap for the proposed unit cell functions as a short state because of the low resistivity of the GeTe films. As a result, the electric field is distributed in both ends of the circular pattern for the proposed absorber as shown in Figure 5b.

## 3. Fabrication Process

**Figure 6:**
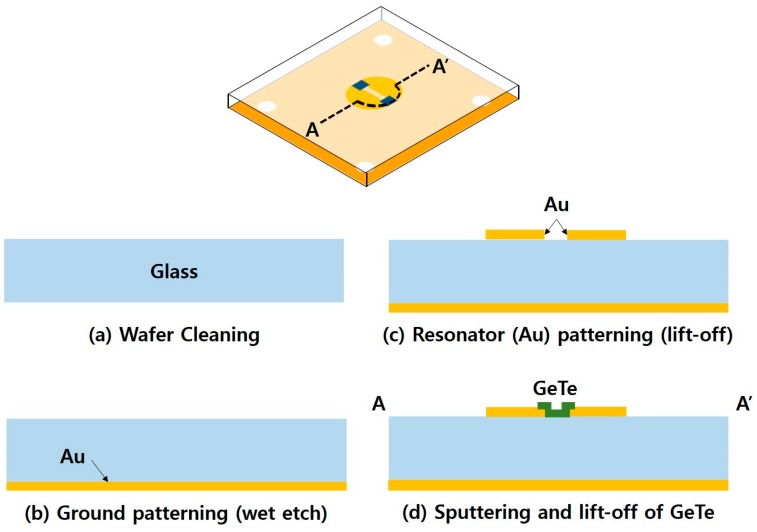
Fabrication process for the proposed absorber (cross-sectional view along line A–A’).

**Figure 7:**
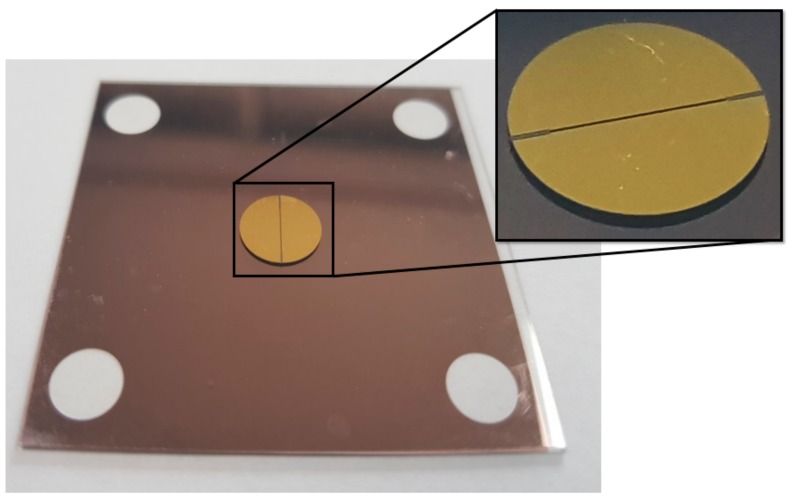
Photograph of fabricated absorber sample.

The overall fabrication process for the proposed absorber is illustrated in Figure 6. A 4-inch, 525-μm-thick borosilicate glass wafer is used as the substrate for the absorber. The wafer is cleaned in a 4:1 mixture of sulfuric acid (H_2_SO_4_) and hydrogen peroxide (H_2_O_2_) to remove contaminants. On the backside of the wafer, a chromium/gold (10 nm/300 nm) layer is deposited with an E-beam evaporator and patterned by wet chemical etching using positive photoresist to form a ground layer for the absorber. Next, an image reversal photoresist (AZ5214E, AZ Electronic Material, Luxembourg) is patterned on the topside of the wafer to form a lift-off mold. Another chromium/gold layer with the same thickness is E-beam evaporated and patterned through lift-off of the photoresist in acetone to form the resonators of the absorber. The lift-off mold for the GeTe switching element is patterned at the designated location using the same process, and then a 100 nm-thick GeTe layer is deposited by RF sputtering using a Ge_50_Te_50_ sputtering target of 99.99% purity. The sputtering chamber is set to a pressure of 15 mTorr with a 150 sccm flow of argon gas and an applied RF power of 150 W. Finally, the GeTe switching element is formed through lift-off of the photoresist. Figure 7 shows a photograph of the fabricated absorber sample. The final sample size after dicing is 41.3 mm × 41.3 mm, and four-hole patterns with a radius of 2.15 mm are placed at the corners of the sample to facilitate easy alignment of the sample to the waveguide for measurement.

## 4. Experimental Verification

**Figure 8:**
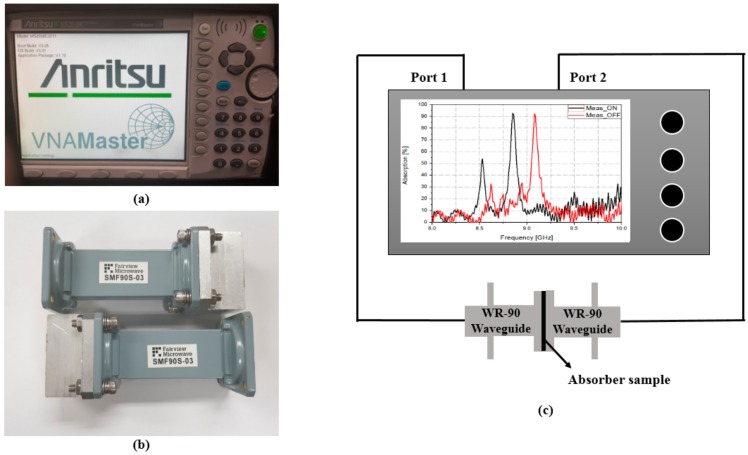
Measurement setup to verify the experiment (**a**) ANRITSU MS2038C Vector Network Analyzer (**b**) WR-90 X-band waveguide (**c**) waveguide measurement setup.

**Figure 9:**
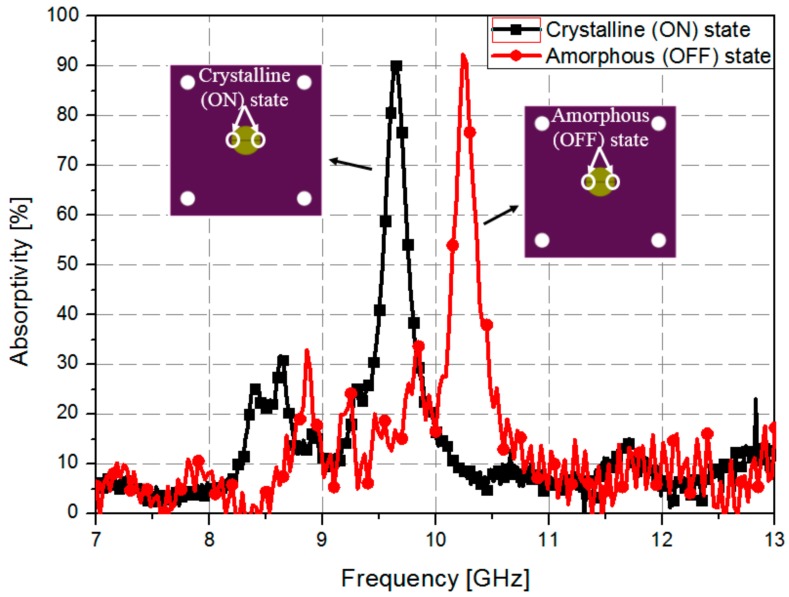
Measured absorptivity versus a frequency at different states of the GeTe.

To measure the characteristics of the fabricated sample, we used an ANRITSU vector network analyzer (VNA), and two WR-90 waveguides. Figure 8 shows the measurement setup used to verify the experiment. The prototype sample was placed between two WR-90 waveguides. The WR-90 waveguide has an aperture dimension of 22.86 mm × 10.16 mm and an operating frequency ranging from 8.0 GHz to 12.4 GHz. We measured only the reflection coefficient to calculate the absorptivity because the bottom side of the sample was completely covered with a gold layer, which prevents the wave from passing through the absorber. The sample was measured using the following procedure. First, the reflection coefficient of the fabricated absorber sample with the as-deposited amorphous GeTe (without heat treatment) thin film was measured at room temperature. Afterward, the sample was heated to 250 °C on a hot plate and kept at this temperature for 30 min to crystallize the GeTe, and the reflection coefficient was measured again. The absorptivity was calculated from the measured S-parameters based on Equation (1). Figure 9 shows the measured absorptivity versus the frequency of GeTe in different states. The absorption frequency changes from 10.23 GHz in the as-deposited amorphous state to 9.6 GHz when the GeTe transitions to the crystalline state at 250 °C, respectively. In addition, the absorptivity was calculated to be 91% and 92% each state, respectively.

The current device platform is simply designed to compare the results of the as-deposited amorphous OFF state and thermally treated crystalline ON state to show the potential of GeTe as a switchable device utilizing an easily accessible waveguide measurement setup. For multiple use of the device in practical applications, opposite phase transition from the crystalline to amorphous state is required. This can be made by utilizing the resonator elements as electrodes and applying a DC pulse with a short trailing edge for melting and rapid quench cooling to amorphize the material, as is done in many microwave switch applications using GeTe. Table 1 shows the comparison table of the proposed frequency reconfigurable absorber with other frequency reconfigurable metamaterial absorbers using VO_2_. The proposed absorber shows a slightly higher tuning ratio (TR) compared to other absorbers using VO_2_.

## 5. Conclusions

In this study, we proposed a thermally frequency switchable electromagnetic absorber using GeTe phase change material. A circular pattern with a gap was proposed and two GeTe films were placed at the edges of the gap to maximize the frequency shift. The performance of the proposed absorber was numerically and experimentally demonstrated. Measurement results show that the absorption frequency changes from 10.23 GHz to 9.6 GHz with 92% and 91% absorptivity, respectively, when the temperature is increased from 25 °C to 250 °C, respectively. The proposed electromagnetic absorber can be potentially used for not only frequency switchable absorption but also wireless high-temperature thermal shock detecting or protection device applications, where non-volatile bi-stable operation of GeTe can be effectively utilized.

## Figures and Tables

**Table 1:** Comparison table of the proposed frequency reconfigurable absorber using GeTe and other frequency reconfigurable absorbers using VO_2_.

Reference Paper	Tuning Technology	Lowest Frequency (*f*_low_) [GHz]	Highest Frequency (*f*_high_) [GHz]	TR ^(1)^
[45]	Vanadium Oxide	9.0	9.6	1.06
[44]	Vanadium Oxide	9.36	9.98	1.06
Proposed work	Germanium Telluride	9.4	10.51	1.11

^(1)^ TR = *f*_high_/*f*_low_.
